# A New Diagnostic Model to Distinguish Kawasaki Disease From Other Febrile Illnesses in Chongqing: A Retrospective Study on 10,367 Patients

**DOI:** 10.3389/fped.2020.533759

**Published:** 2020-11-12

**Authors:** Zhilin Huang, Xu-Hai Tan, Haolin Wang, Bo Pan, Tie-Wei Lv, Jie Tian

**Affiliations:** ^1^Department of Heart, Ministry of Education Key Laboratory of Child Development and Disorders, National Clinical Research Center for Child Health and Disorders, China International Science and Technology Cooperation Base of Child Development and Critical Disorders, Chongqing Key Laboratory of Pediatrics, Children's Hospital of Chongqing Medical University, Chongqing, China; ^2^Department of Pediatric, People's Hospital of Hongan, Hubei, China; ^3^College of Medical Informatics, Chongqing Medical University, Chongqing, China

**Keywords:** kawasaki disease, children, febrile illnesses, diagnostic model, independent predictors

## Abstract

**Objective:** Kawasaki disease (KD) is one of the most prevailing vasculitis among infants and young children, and has become the leading cause of acquired heart disease in childhood. Delayed diagnosis of KD can lead to serious cardiovascular complications. We sought to create a diagnostic model to help distinguish children with KD from children with other febrile illnesses [febrile controls (FCs)] to allow prompt treatment.

**Methods:** Significant independent predictors were identified by applying multivariate logistic regression analyses. A new diagnostic model was constructed and compared with that from diagnostic tests created by other scholars.

**Results:** Data from 10,367 patients were collected. Twelve independent predictors were determined: a lower percentage of monocytes (%MON), phosphorus, uric acid (UA), percentage of lymphocyte (%LYM), prealbumin, serum chloride, lactic dehydrogenase (LDH), aspartate aminotransferase: alanine transaminase (AST: ALT) ratio, higher level of globulin, gamma-glutamyl transpeptidase (GGT), platelet count (PLT), and younger age. The AUC, sensitivity, and specificity of the new model for cross-validation of the KD diagnosis was 0.906 ± 0.006, 86.0 ± 0.9%, and 80.5 ± 1.5%, respectively. An equation was presented to assess the risk of KD, which was further validated using KD (*n* = 5,642) and incomplete KD (*n* = 809) cohorts.

**Conclusions:** Children with KD could be distinguished effectively from children with other febrile illnesses by documenting the age and measuring the level of %MON, phosphorus, UA, globulin, %LYM, prealbumin, GGT, AST:ALT ratio, serum chloride, LDH, and PLT. This new diagnostic model could be employed for the accurate diagnosis of KD.

## Introduction

Kawasaki disease (KD) is a vasculitis of unknown etiology that, in general, occurs in childhood and is the most common cause of acquired heart disease (1). The incidence of KD is highest in children who live in East Asia or who are of Asian ancestry living in other parts of the world (2–5). KD incidence in underdeveloped regions and countries is not known as few cases are reported (e.g., in Southeast Asia), which may be related to the lower level of diagnosis.

KD can cause cardiovascular complications. In particular, coronary-artery aneurysms (CAAs) develop in about 15–25% of children who have not been treated for KD (6). These CAAs are associated mainly with occlusion of coronary arteries and cardiac ischemia, which can result in increased morbidity and even mortality.

The prevalence of CAA development in KD and related morbidity and mortality has decreased significantly as a result of treatment with high-dose intravenous immunoglobulin (IVIG) (7, 8). Early diagnosis is the most vital factor in achieving optimal treatment outcomes.

However, rapid discrimination of KD from other febrile illnesses is difficult, which leads to delays in the diagnosis of KD and treatment with IVIG. Diagnosis beyond 10 days of fever has been suggested to result in an increased prevalence of CAAs by 2.8- to 7.1-fold (9, 10). Patients who fail to meet the principal clinical findings for a diagnosis of KD (referred to as “incomplete KD”) may develop CAAs.

Diagnosis of KD in the earliest phase after symptom onset is crucial and it is important to initiate treatment to lower the risk of CAAs (11). However, timely identification is challenging because diagnosis is based on clinical findings and nonspecific laboratory testing (12, 13). A specific diagnostic approach for patients with KD is lacking. The diagnosis of KD according to the criteria established by Tomisaku Kawasaki in 1967 is based on a constellation of clinical features (14). The clinical features of KD overlap with those of many other common childhood illnesses, such as infection by echoviruses, adenoviruses (15), Epstein–Barr virus (EBV), and measles. These viral illnesses share many of the signs of mucocutaneous inflammation and closely mimic KD. There is, therefore, an urgent need for sensitive and specific diagnostic tests to discriminate KD from other conditions that also cause prolonged fever in children.

Numerous studies have reported some discrimination between KD and other febrile illnesses based on certain laboratory parameters, but none have been validated (16–18). The major issue with those studies has been the selection of febrile controls (FCs), which might not represent the population of patients who could be confused with KD patients. Another issue has been the use of different models for prediction from different populations, which may not be sufficiently accurate and sensitive in Chinese populations (19). In addition, a common limitation of those reports was a small study cohort.

This retrospective study aimed to identify significant predictors and establish a new diagnostic model to differentiate children with KD from FCs. We reviewed the data from 10,367 patients from Chongqing City in China. We compared our data with results from studies by Falcini et al. (16), Barone et al. (19), Okada et al. (18), Song et al. (20), and Ling et al. (21) with regard to predictive ability, sensitivity, and specificity.

## Materials and Methods

### Ethical Approval of the Study Protocol

The study protocol were approved by the Ethics Committee of the Children's Hospital Affiliated to Chongqing Medical University (Chongqing, China). Written informed consent from the parents of children was not required. The study was undertaken in accordance with the Declaration of Helsinki 1964 and its later amendments.

### Study Design

We evaluated (retrospectively) the clinical findings of consecutive KD patients and FCs (who shared some features of KD) treated from October 2007 to December 2017 in Chongqing Children's Hospital (Chongqing, China). These patients were divided into two groups: KD and FCs.

The diagnostic criteria for KD in our hospital are in accordance with those set by the American Heart Association (22). These diagnostic criteria include ≥5 days of fever accompanied by four or five of the following clinical findings: (i) bilateral conjunctival injection; (ii) changes in the oral mucous membranes; (iii) changes in the peripheral extremities; (iv) polymorphous rash; (v) cervical lymphadenopathy. The inclusion criterion was KD as the main diagnosis upon hospital discharge. Patients who received IVIG treatment in other medical institutions before hospital admission were excluded from our study.

FCs had a documented fever (≥38.0°C) accompanied by at least one of the following clinical signs of KD: (i) skin rash; (ii) conjunctival injection; (iii) enlargement of cervical lymph nodes; (iv) changes in the peripheral extremities; (v) pharyngeal abnormalities (21). We also compared incomplete KD and FCs to further validate our model. “Incomplete KD” were said to occur if there were ≤ 3 of the clinical findings of KD.

### Data Collection

A total of 10,367 people met the inclusion criteria and were enrolled in our study to develop the model. There were 5,642 cases in the KD group (54.42%) and 4,725 casesin the FCs group (45.58%). The data of 809 cases with incomplete KD were collected to further validate the performance of the developed model.

Data before initial IVIG treatment were collected: age (months); sex; white blood cell count (WBC); platelet distribution width (PDW); platelet count (PLT); mean platelet volume (MPV); red blood cell count (RBC); hemoglobin (HB); packed cell volume (PCV); red blood cell distribution width (RDW); total red blood cell distribution width (RDWa); erythrocyte morphology; mean corpuscular hemoglobin (MCH); mean corpuscular volume (MCV); platelet-large-cell ratio (P-LCR); total number of lymphocytes; total number of monocytes; total number of neutrophils; percentage of lymphocytes (%LYM); thrombocytosis; percentage of neutrophils (%NEU); leucocyte morphology; percentage of monocytes (%MON); hematuria; urinary vitamin C; urinary sugar; urinary protein; urinary bilirubin; urine transparency; ovum (stool); phagocytes in stool; red blood cells in stool; gamma-glutamyl transpeptidase (GGT); alkaline phosphatase (ALP); lactic dehydrogenase (LDH); aspartate aminotransferase (AST); alanine transaminase (ALT); AST:ALT ratio; direct bilirubin (DBIL); albumin; prealbumin; total protein (TP); total bilirubin (TBIL); globulin; ketone body (KET); creatinine; bile acid (BA); blood urea nitrogen (BUN); uric acid (UA); C-reactive protein (CRP); phosphorus; and serum levels of sodium, potassium, magnesium, chloride, and calcium upon hospital admission.

If there were more than two laboratory reports before the initial IVIG treatment with regard to routine blood analyses, kidney function, routine urinalyses, liver function, routine stool analyses, CRP level, and electrolytes, we used the reports with the highest values of WBC, %NEU, ALT, AST, BUN, CRP and lowest levels of TP, serum chloride, and albumin (23).

### Statistical Analyses

De-identified clinical laboratory findings were extracted from electronic medical records (EMRs) for comparison between the KD group and the FCs group. For variables with a missing detection rate <25%, we undertook multiple imputations by chained equations (MICE) (24). MICE is the principal method to address the problem of missing data and was employed to reduce bias in our study. The adopted method for MICE was linear regression, and the number of multiple imputations and the number of iterations were 5 and 10, respectively. Data are the mean ± standard deviation (SD) for continuous data or as a percentage for categorical data (Table 1).

**Table 1:** Univariate analysis comparison of the KD group and FCs group.

**Variable**	**KD group**		**FCs group**		***P*-value**
	***N***	**Mean ± SD/Counts (%)**	***N***	**Mean ± SD/Counts (%)**	
**Blood test**					
Red blood cell count, 1,012/L	4,593	3.97 ± 0.45	4,379	4.26 ± 0.54	<0.001
Absolute value of Red blood cell Distribution, fL	4,244	40.49 ± 4.52	4,174	41.56 ± 5.92	<0.001
Red blood cell distribution width, %	4,559	13.95 ± 1.78	4,368	14.24 ± 2.00	0.001
Packed cell volume, %	4,592	31.98 ± 3.54	4,379	34.61 ± 4.06	<0.001
Erythrocyte morphology (abnormal)^*^	4,405	291 (0.066)	4,346	322 (0.074)	<0.001
Mean platelet volume, fL	4,358	9.91 ± 1.06	4,161	10.19 ± 1.11	<0.001
Platelet distribution width, fL	4,430	11.48 ± 2.23	4,159	11.84 ± 2.41	<0.001
Thrombocytocrit, %	4,222	0.44 ± 0.50	3,923	0.39 ± 0.54	<0.001
Platelet count, 109/L	4,593	384.53 ± 163.33	4,379	308.46 ± 146.99	<0.001
White blood cell, 109/L	4,592	15.11 ± 6.31	4,379	10.87 ± 6.91	<0.001
Mean Corpuscular Hemoglobin, pg	4,440	26.29 ± 2.11	4,378	26.62 ± 2.61	<0.001
Mean corpuscular volume	4,593	80.79 ± 6.28	4,379	81.70 ± 2.29	<0.001
Absolute value of lymphocyte	4,329	3.63 ± 2.02	4,257	4.09 ± 2.77	0.016
Leucocyte morphology (abnormal)^*^	4,522	55 (0.012)	4,180	86 (0.021)	<0.001
Percentage of lymphocyte	4,592	0.26 ± 0.14	4,379	0.42 ± 0.20	<0.001
Absolute value of neutrophil	4,481	10.73 ± 5.71	4,260	6.14 ± 5.93	<0.001
Percentage of neutrophil	4,593	0.69 ± 0.16	4,379	0.52 ± 0.22	<0.001
Absolute value of monocyte	4,196	0.42 ± 0.65	4,082	0.39 ± 0.29	<0.001
Percentage of monocyte	4,399	0.03 ± 0.02	4,314	0.04 ± 0.02	<0.001
Platelet-large-cell ratio, %	4,174	24.24 ± 8.20	3,870	26.24 ± 8.80	0.001
Hemoglobin, g/l	4,593	104.14 ± 11.61	4,379	112.81 ± 14.32	<0.001
**Urine test**					
Blood urine (positive)^*^	4,932	302 (0.061)	4,230	223 (0.053)	<0.001
Vitamin C (positive)^*^	4,932	2,870 (0.582)	4,256	1,951 (0.458)	<0.001
Urine sugar (positive)^*^	5,092	737 (0.145)	4,236	286 (0.068)	<0.001
Urine protein (positive)^*^	5,094	575 (0.113)	4,256	168 (0.039)	<0.001
Urobilirubin (positive)^*^	5,094	132 (0.026)	4,256	21 (0.005)	0.001
The transparency of the urine (positive)^*^	4,931	570 (0.116)	4,252	243 (0.057)	<0.001
**Stool test**					
Ovum (positive)^*^	4,990	0	4,276	1 (<0.001)	<0.001
Red blood cell (positive)^*^	4,990	66 (0.013)	4,276	43 (0.010)	<0.001
Phagocyte (positive)^*^	4,990	2 (<0.001) (0.00)	4,276	0	<0.001
**Biochemical test**					
Gamma- glutamyl transpeptidase, U/L	5,043	86.14 ± 111.70	4,286	34.43 ± 72.89	<0.001
Alanine transaminase, IU/L	5,043	69.32 ± 100.90	4,285	43.54 ± 120.35	<0.001
Aspartate aminotransferase, IU/L	5,271	50.53 ± 90.22	4,286	63.02 ± 205.80	<0.001
Lactic dehydrogenase, IU/L	5,272	300.45 ± 151.70	4,286	398.71 ± 534.56	<0.001
Alkaline phosphatase, IU/L	5,043	182.00 ± 121.16	4,286	184.31 ± 101.62	0.007
AST:ALT ratio	5,042	1.17 ± 0.83	4,284	1.05 ± 0.78	<0.001
Direct bilirubin, umol/L	4,678	5.55 ± 10.79	3,844	3.02 ± 5.67	<0.001
Total bilirubin, umol/L	5,037	10.77 ± 14.25	4,284	9.69 ± 18.51	<0.001
Total Protein, g/L	5,043	60.01 ± 7.40	4,289	62.95 ± 7.63	<0.001
Albumin, g/L	5,043	36.76 ± 4.96	4,289	41.38 ± 5.67	<0.001
Prealbumin, mg/L	4209	65.50 ± 41.73	3,694	124.88 ± 54.58	<0.001
Globulin, g/L	5,043	23.25 ± 5.98	4,289	21.57 ± 6.02	<0.001
Creatinine, umol/L	4,894	26.27 ± 16.43	4075	29.39 ± 21.49	<0.001
Blood urea nitrogen, mmol/L	4,892	2.90 ± 1.52	4,075	3.46 ± 2.38	<0.001
Ketone body^*^	5,094	0.49 ± 0.97	4,256	0.39 ± 0.88	<0.001
Bile acid	4,226	22.15 ± 43.60	3,790	12.40 ± 23.76	<0.001
Uric acid, umol	4,885	210.20 ± 83.58	4,074	259.66 ± 115.96	<0.001
**Inflammatory factor**					
C-reactive protein, mg/L	4,421	60.08 ± 52.85	4,256	23.03 ± 43.19	<0.001
**Ion**					
Serum phosphorus, mmol/L	4,858	1.30 ± 0.30	4,131	1.50 ± 0.35	<0.001
Serum sodium, mmol/L	4,861	137.19 ± 3.26	4,151	138.34 ± 3.54	<0.001
Serum potassium, mmol/L	4,861	4.22 ± 0.68	4,149	4.40 ± 0.65	<0.001
Serum magnesium, mmol/L	4,859	0.92 ± 0.11	4,131	0.93 ± 0.11	<0.001
Serum chloride, mmol/L	4,859	101.20 ± 3.76	4,132	103.28 ± 4.37	<0.001
Serum calcium, mmol/L	4,499	2.29 ± 0.16	4,016	2.26 ± 0.20	<0.001
**Demographics**					
Age, month	5,642	31.75 ± 25.11	4,725	42.35 ± 42.34	<0.001
Sex (male)^*^	5,641	3,943 (0.70)	4,725	2,785 (0.59)	<0.001

ALT, Alanine transaminase; AST, Aspartate aminotransferase;

* *for categorical variables; N, number of samples; SD, standard deviation; W value for Wilcoxon-Mann-Whitney test; χ2 value for chi-square test*.

One of our challenges was that KD assessment is not very sensitive to individual predictors. To identify significant predictors effectively, data were standardized (rescaled) to have a mean of 0 and an SD of 1. The Mann–Whitney *U*-test was carried out for comparison of continuous data. Categorical data were assessed using the chi-square test for comparison between the two groups. For all analyses, *P* < 0.05 was considered significant. Selected data that were significantly different between the two groups were entered into multivariate analyses. To develop a reliable prediction model for the KD diagnosis, we divided the dataset into five subgroups randomly. One of the five subgroups was used as the test set and the remaining four subgroups were used to form the training set each time, and the experiments were repeated five times (known as 5-fold cross-validation). The least absolute shrinkage and selection operator (LASSO) regression model were applied for further feature selection using the significantly different indicators obtained by the univariate analysis. Finally, we developed the diagnostic model based on multivariate logistic regression analysis. The odds ratio (OR) with a 95% confidence interval (CI) was calculated to determine the score of an independent predictor and establish a new prediction model. We did not carry out the Hosmer–Lemeshow test because it can lead to misleadingly significant values with large sample sizes. The predictive performance of the proposed model was evaluated using the receiver operating characteristic (ROC) curve and the area under the ROC curve (AUC). We constructed an equation to increase the usefulness of the individual risk probability of KD diagnosis that could be applied in clinical practice. Statistical analyses were conducted using Python for Statistical Computing.

## Results

### Comparison Between the KD Group and FCs Group by Univariate Analysis

Table 1 shows the clinical/laboratory findings in the two groups using univariate analysis. The level of 24 variables of the KD group was significantly higher than that of the FCs group: thrombocytosis; PLT; WBC; total number of neutrophils; %NEU; total number of monocytes; hematuria; vitamin C in urine; sugar in urine; protein in urine; bilirubin in urine; urine transparency; phagocytes in stool; red blood cells in stools; GGT; ALT; DBIL; TBIL; globulin; KET; BA; CRP; serum calcium.

The level of 32 variables was significantly lower in the KD group than that in the FCs group: RDWa; RDW; PCV; abnormal erythrocyte morphology; MPV; RBCs; PDW; MCH; MCV; total number of lymphocytes; abnormal leukocyte morphology; %LYM; %MON; P-LCR; HB; ovum in stools; AST; AST:ALT ratio; LDH; ALP; TP; albumin; prealbumin; creatinine; BUN; UA; phosphorus; age; serum levels of sodium, chloride, potassium, and magnesium.

Patients in the KD group were predominantly male and younger than those in the FCs group.

### Independent Predictors and Diagnostic Model for KD

For multivariate logistic regression analyses, we selected significant variables derived from the univariate analysis through LASSO constraints to balance accuracy and simplicity. Fifteen variables (one demographic variable and 14 laboratory variables) were identified by “tuning” of the hyper-parameter lambda. Among the 15 variables, however, 12 variables were significant and were applied to multivariate logistic regression analyses. No significant difference was observed for the level of CRP, albumin, or HB (Table 2). Multivariate logistic regression analysis identified significant independent predictors for the KD group to be: lower levels of %MON, phosphorus, UA, %LYM, prealbumin, AST:ALT ratio, serum chloride, and LDH; higher levels of globulin, GGT, and PLT; younger age. Table 3 shows the OR (95%CI) values of those predictors.

**Table 2:** The OR (95%CI) values of the independent predictors for the KD diagnosis.

**Multiple logistic regression analysis after LASSO (5-fold cross validation)**										
**Predictors**	**Group 1**		**Group 2**		**Group 3**		**Group 4**		**Group 5**	
	**OR value (95%CI)**	***P*****-value**	**OR value (95%CI)**	***P*****-value**	**OR value (95%CI)**	***P*****-value**	**OR value (95%CI)**	***P*****-value**	**OR value (95%CI)**	***P*****-value**
%MON	0.620 (0.575–0.669)	<0.001	0.616 (0.571–0.665)	<0.001	0.621 (0.576–0.670)	<0.001	0.648 (0.601–0.700)	<0.001	0.657 (0.610–0.708)	<0.001
CRP	1.085 (0.996–1.182)	0.062	1.070 (0.985–1.163)	0.11	1.099 (1.008–1.198)	0.032	1.108 (1.018–1.207)	0.018	1.015 (0.933–1.103)	0.731
Phosphorus	0.659 (0.608–0.714)	<0.001	0.662 (0.612–0.717)	<0.001	0.686 (0.634–0.743)	<0.001	0.689 (0.636–0.746)	<0.001	0.651 (0.601–0.706)	<0.001
UA	0.748 (0.690–0.811)	<0.001	0.728 (0.672–0.787)	<0.001	0.729 (0.673–0.789)	<0.001	0.704 (0.650–0.762)	<0.001	0.728 (0.672–0.788)	<0.001
Globulin	1.484 (1.373–1.605)	<0.001	1.492 (1.382–1.611)	<0.001	1.479 (1.368–1.598)	<0.001	1.488 (1.377–1.609)	<0.001	1.509 (1.395–1.632)	<0.001
Albumin	0.956 (0.873–1.047)	0.332	0.939 (0.860–1.026)	0.164	0.995 (0.910–1.088)	0.906	0.938 (0.857–1.027)	0.165	0.915 (0.836–1.002)	0.054
%LYM	0.499 (0.458–0.544)	<0.001	0.496 (0.455–0.541)	<0.001	0.491 (0.451–0.535)	<0.001	0.485 (0.445–0.529)	<0.001	0.478 (0.439–0.522)	<0.001
Prealbumin	0.323 (0.290–0.360)	<0.001	0.364 (0.329–0.404)	<0.001	0.335 (0.301–0.373)	<0.001	0.357 (0.322–0.397)	<0.001	0.361 (0.325–0.401)	<0.001
HB	0.934 (0.863–1.010)	0.088	0.911 (0.843–0.985)	0.019	0.932 (0.862–1.009)	0.083	0.923 (0.853–0.998)	0.045	0.918 (0.848–0.994)	0.036
GGT	1.245 (1.142–1.358)	<0.001	1.296 (1.186–1.416)	<0.001	1.317 (1.199–1.446)	<0.001	1.293 (1.182–1.415)	<0.001	1.366 (1.238–1.508)	<0.001
AST:ALT ratio	0.579 (0.530–0.633)	<0.001	0.581 (0.532–0.634)	<0.001	0.589 (0.538–0.645)	<0.001	0.566 (0.518–0.619)	<0.001	0.571 (0.523–0.624)	<0.001
Chloride	0.757 (0.700–0.819)	<0.001	0.790 (0.736–0.849)	<0.001	0.765 (0.713–0.821)	<0.001	0.735 (0.684–0.790)	<0.001	0.737 (0.686–0.792)	<0.001
LDH	0.309 (0.254–0.374)	<0.001	0.368 (0.306–0.443)	<0.001	0.358 (0.297–0.431)	<0.001	0.409 (0.341–0.490)	<0.001	0.353 (0.292–0.425)	<0.001
PLT	1.567 (1.448–1.696)	<0.001	1.532 (1.417–1.657)	<0.001	1.547 (1.430–1.673)	<0.001	1.582 (1.462–1.712)	<0.001	1.526 (1.408–1.653)	<0.001
AGE	0.435 (0.401–0.472)	<0.001	0.446 (0.412–0.484)	<0.001	0.449 (0.415–0.487)	<0.001	0.455 (0.419–0.493)	<0.001	0.441 (0.406–0.478)	<0.001

*OR, odds ratio; CI, confidence interval; LASSO, least absolute shrinkage, and selection operator; %MON, percentage of monocyte; CRP, C-reactive protein; UA, uric acid; %LYM, percentage of lymphocyte; HB, hemoglobin; GGT, gamma-glutamyl transpeptidase; AST, aspartate aminotransferase; ALT, alanine transaminase; LDH, lactic dehydrogenase; PLT, platelet count*.

**Table 3:** The OR (95%CI) values of the independent predictors for the KD diagnosis.

**Multiple logistic regression analysis using the 12 indicators with statistical significance (5-fold cross validation)**										
**Predictors**	**Group 1**		**Group 2**		**Group 3**		**Group 4**		**Group 5**	
	**OR value (95%CI)**	***P*****-value**	**OR value (95%CI)**	***P*****-value**	**OR value (95%CI)**	***P*****-value**	**OR value (95%CI)**	***P*****-value**	**OR value (95%CI)**	***P*****-value**
%MON	0.614 (0.569–0.662)	<0.001	0.608 (0.563–0.655)	<0.001	0.614 (0.569–0.662)	<0.001	0.639 (0.592–0.689)	<0.001	0.649 (0.603–0.699)	<0.001
Phosphorus	0.650 (0.600–0.705)	<0.001	0.654 (0.604–0.708)	<0.001	0.682 (0.630–0.738)	<0.001	0.680 (0.628–0.736)	<0.001	0.641 (0.592–0.695)	<0.001
UA	0.743 (0.686–0.806)	<0.001	0.722 (0.667–0.781)	<0.001	0.727 (0.672–0.787)	<0.001	0.699 (0.646–0.757)	<0.001	0.722 (0.666–0.781)	<0.001
Globulin	1.509 (1.397–1.629)	<0.001	1.521 (1.410–1.641)	<0.001	1.499 (1.388–1.619)	<0.001	1.520 (1.407–1.641)	<0.001	1.535 (1.420–1.658)	<0.001
%LYM	0.480 (0.442–0.520)	<0.001	0.477 (0.440–0.518)	<0.001	0.472 (0.436–0.512)	<0.001	0.461 (0.425–0.501)	<0.001	0.468 (0.431–0.508)	<0.001
Prealbumin	0.304 (0.275–0.335)	<0.001	0.340 (0.309–0.373)	<0.001	0.320 (0.290–0.353)	<0.001	0.331 (0.300–0.364)	<0.001	0.337 (0.306–0.371)	<0.001
GGT	1.266 (1.161–1.380)	<0.001	1.323 (1.211–1.445)	<0.001	1.329 (1.211–1.459)	<0.001	1.322 (1.208–1.446)	<0.001	1.401 (1.270–1.545)	<0.001
AST:ALT ratio	0.574 (0.526–0.626)	<0.001	0.573 (0.525–0.625)	<0.001	0.583 (0.533–0.638)	<0.001	0.557 (0.510–0.609)	<0.001	0.564 (0.517–0.616)	<0.001
Chloride	0.752 (0.696–0.813)	<0.001	0.784 (0.730–0.843)	<0.001	0.762 (0.710–0.818)	<0.001	0.729 (0.679–0.783)	<0.001	0.735 (0.684–0.789)	<0.001
LDH	0.315 (0.260–0.382)	<0.001	0.378 (0.314–0.454)	<0.001	0.361 (0.300–0.435)	<0.001	0.417 (0.348–0.499)	<0.001	0.359 (0.298–0.433)	<0.001
PLT	1.599 (1.481–1.728)	<0.001	1.569 (1.453–1.695)	<0.001	1.577 (1.460–1.703)	<0.001	1.621 (1.501–1.751)	<0.001	1.559 (1.442–1.686)	<0.001
AGE	0.433 (0.399–0.469)	<0.001	0.443 (0.409–0.479)	<0.001	0.447 (0.413–0.483)	<0.001	0.452 (0.417–0.489)	<0.001	0.436 (0.403–0.472)	<0.001

*OR, odds ratio; CI, confidence interval; LASSO, least absolute shrinkage, and selection operator; %MON, percentage of monocyte; UA, uric acid; %LYM, percentage of lymphocyte; GGT, gamma-glutamyl transpeptidase; AST, aspartate aminotransferase; ALT, alanine transaminase; LDH, lactic dehydrogenase; PLT, platelet count*.

We obtained a model as shown in Equation (1):

$$\begin{array}{l} \text{ln}\left(\text{P}/\left(1-\text{P}\right)\right)=0.211+\left(-0.471\right)\times \%\text{MON}+\left(-0.414\right)\\ \;\times \text{phosphorus}+\left(-0.325\right)\times \text{UA}+\left(0.416\right)\\ \;\times \text{globulin}+\left(-0.751\right)\times \%\text{LYM}+\left(-1.121\right)\\ \;\times \text{prealbumin}+\left(0.283\right)\times \text{GGT}+\left(-0.562\right)\\ \;\times \text{AST}:\text{ALTratio}+\left(-0.285\right)\times \text{chloride}\\ \;+\left(-1.009\right)\times \text{LDH}+\left(0.461\right)\times \text{PLT}\\ \;+\left(-0.817\right)\times \text{age}\left(\text{inmonths}\right) \end{array}$$

where P is the expected probability that the diagnosis is KD.

Hence, we could determine the individual-risk probability of the KD diagnosis. The coefficients represent the contribution of the variables in Equation (1). Taking the GGT level as an example and assuming that the other items are unchanged, the OR of having the KD diagnosis increases by 26.6% (OR – 1 = 1.266 – 1 = 0.266) with an increase of one SD (one rescaled unit) in the GGT level. The greater the positive coefficient of the level of globulin, the GGT level, and PLT level would increase the possibility of KD diagnosis. The greater the negative coefficient in the level of %MON, phosphorus, UA, %LYM, prealbumin, AST:ALT ratio, chloride, LDH and age would decrease the OR of the KD diagnosis. Taking a patient with confirmed KD as an example, the indicators would be: PLT = 801 ×10^9^/L (normal range, 100–380); %LYM = 0.11 (0.3–0.6); %MON = 0.06 (0.02–0.08); GGT = 150 U/L (0–25); globulin = 20.2 g/L (15.3–35); phosphorus = 0.74 mmol/L (1.29–2.26); UA = 128 μmol/L (100–410), prealbumin = 60 mg/L (100–300); AST:ALT ratio = 0.36 (0.23–2.47); chloride = 95.9 mmol/L (98–107); LDH = 300 U/L (110–330); age = 25 months.

McFadden's *R*^2^ was 0.431 ± 0.005 for our model. The sensitivity, specificity, and AUC values of the 5-fold cross-validation are shown in Table 4. The AUC, sensitivity, and specificity of our diagnostic model for the KD diagnosis was 0.906 ± 0.006, 86.0 ± 0.9%, and 80.5 ± 1.5%, respectively.

**Table 4:** The diagnostic capabilities of the new model and the previous studies.

	**AUC**	**Sensitivity**	**Specificity**
The new model	0.906 ± 0.006	0.860 ± 0.009	0.805 ± 0.015
Falcini et al.	0.791 ± 0.012	0.784 ± 0.012	0.686 ± 0.018
Okada et al.	0.785 ± 0.014	0.779 ± 0.013	0.702 ± 0.022
Barone et al.	0.798 ± 0.017	0.787 ± 0.022	0.700 ± 0.012
Xiu-Yu et al.	0.793 ± 0.014	0.780 ± 0.011	0.704 ± 0.018
Ling et al.	0.724 ± 0.013	0.758 ± 0.010	0.594 ± 0.019

*AUC, area under the curve*.

The logistic model for the identified variables without standardization used to support further investigations is shown in Equation (2).

$$\begin{array}{l} \text{Ln}\left(\text{P}/\left(1-\text{P}\right)\right)=13.534+\left(-26.224\right)\times \%\text{MON}+\left(-1.227\right)\\ \;\times \text{phosphorus}+\left(-0.003\right)\times \text{UA}+\left(0.069\right)\\ \;\times \text{globulin}+\left(-3.905\right)\times \%\text{LYM}\%+\left(-0.020\right)\\ \;\times \text{prealbumin}+\left(0.003\right)\times \text{GGT}+\left(-0.552\right)\\ \;\times \text{AST}:\text{ALTratio}+\left(-0.068\right)\times \text{chloride}\\ \;+\left(-0.003\right)\times \text{LDH}+\left(0.003\right)\times \text{PLT}\\ \;+\left(-0.024\right)\times \text{age}\left(\text{months}\right) \end{array}$$

**Figure 1:**
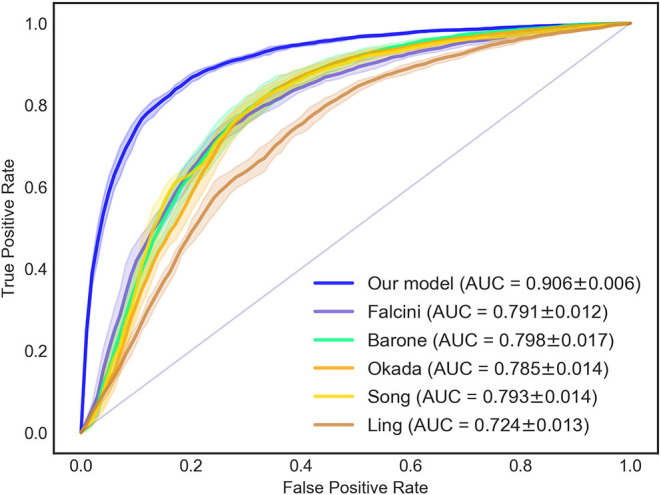
ROC and AUC of the diagnostic models for KD diagnosis. The AUC of the new KD diagnostic prediction model was 0.906 ± 0.006. Compared with previous KD diagnosis studies, the AUC value of the new model was higher than the methods of Falcini (0.791 ± 0.012), Barone (0.798 ± 0.017), Okada (0.785 ± 0.014), Song (0.793 ± 0.014), and Ling (0.724 ± 0.013). ROC, receiver-operator characteristic curves; AUC, area under the curve.

We validated the proposed model (Equation 2) using the collected dataset (cohort of 10,367 patients): a consistent performance was obtained. The ROC curve is shown in Figure 1, and the AUC, sensitivity, and specificity were 0.906 ± 0.006, 86.0 ± 0.9%, and 80.5 ± 1.5%, respectively.

### Comparison Between the New Diagnostic Model and Models Used in Previous Diagnostic Studies

Compared with previous studies in which the KD diagnosis was tested, Figure 1 shows that our model had an AUC (0.906 ± 0.006) that was higher than that obtained in the studies of Falcini et al. (0.791 ± 0.012), Barone et al. (0.798 ± 0.017), Okada et al. (0.785 ± 0.014), Song et al. (0.793 ± 0.014), and Ling et al. (0.724 ± 0.013).

**Figure 2:**
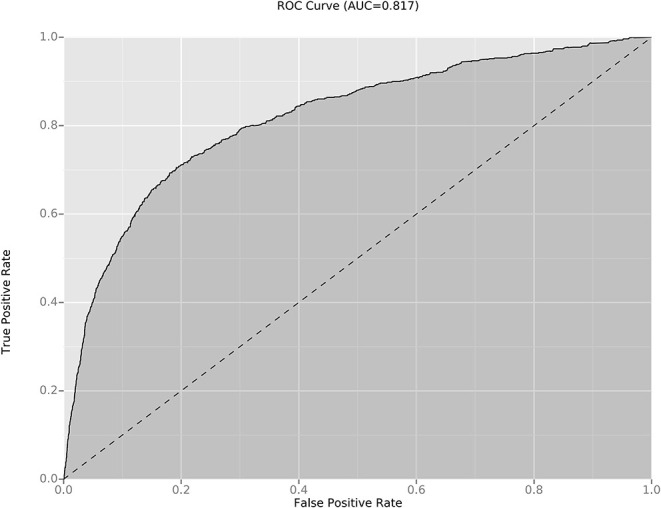
ROC and AUC of the diagnostic models for incomplete KD diagnosis. The AUC value of the new diagnostic model for incomplete KD diagnosis was 0.816. ROC, receiver-operator characteristic curves; AUC, area under the curve.

We compared the model for the KD diagnosis in those previous studies with the KD diagnosis in our cohort: the sensitivity and specificity in our new model were better (Table 4). In addition, a validation dataset (809 patients with incomplete KD) was used to further assess the effectiveness of our new diagnostic model: the AUC was 0.816 (Figure 2). The sensitivity and specificity of this regression model were 70.6 and 80.7%, respectively.

## Discussion

We discovered that a high level of GGT, PLT, and globulin, a low level of %MON, phosphorus, UA, %LYM, prealbumin, AST:ALT ratio, chloride, LDH, and age were independent predictors for the diagnosis of KD. We developed a new model to diagnose KD accurately, with high sensitivity and specificity for the early diagnosis of KD that could be used as the basis of a diagnostic test.

Importantly, we reviewed (retrospectively) 10,367 patients from Chongqing (one of the biggest cities in western China) and built a new model that can be used in the early diagnosis of KD in underdeveloped countries where a poor standard of living, literacy rate, and other socio-economic conditions can be a great challenge.

The KD diagnosis is based mainly on clinical findings and non-specific laboratory indicators. However, several febrile illnesses and KD have similar clinical manifestations: scarlet fever, EBV infection, juvenile idiopathic arthritis, measles, and adenovirus infection. In addition, 15–36.2% of children with KD do not have all the clinical manifestations of KD (incomplete KD), which can lead to misdiagnosis or delayed diagnosis of KD (25). Therefore, our new algorithm for KD diagnosis was validated in patients with incomplete KD (who display atypical findings and constitute a major concern in the diagnosis of a child with a fever of >5-day duration). The AUC of our predictive model was 0.816, which suggests that it is useful and reliable.

For fever patients with the assertive KD diagnosis, the timely initiation of treatment with IVIG can reduce the risk of CAAs significantly. Patients with incomplete KD who do not have the principal clinical features of KD but have a prolonged unexplained fever and inflammation carry an increased risk of CAAs (26). One reason for the increased risk of developing CAAs in atypical KD is a late diagnosis, which usually occurs in patients that do not exhibit all the clinical signs of KD. Given the overlap in clinical presentation with other conditions that also cause a prolonged fever in children (27), initial treatment with a single, high dose of IVIG is likely to be delayed while awaiting exclusion of other febrile illnesses. Furusho et al. (28) and Newburger et al. (7) reported that initial treatment with IVIG within the first 10 days of illness reduced the prevalence of CAAs 5-fold compared with that in children not treated with IVIG. Thus, a specific and sensitive diagnostic test that distinguishes KD from other febrile illnesses accurately would be a huge advance in KD management, reducing needless examinations and inappropriate treatments, and enabling prompt administration of IVIG.

In establishing the FCs group, our aim was to include several illnesses with symptoms that overlap with KD: lymphangitis, exanthema subitum, measles, and other viral illnesses (e.g., adenovirus infection), and childhood inflammatory disorders. The features that we recognized enabled discrimination of KD from other febrile illnesses of childhood and overlapping inflammatory symptoms. Some patients with non-KD disease but with semblable signs could be treated with IVIG. In the absence of pathognomonic features, the diagnosis of KD is reliant on the identification of principal clinical findings and exclusion of other similar diseases with known causes, which leads to a high missed detection rate for the first visit/preliminary diagnosis. Therefore, we used routinely collected electronic medical records (EMRs) data that are available at the early stage of hospitalization to distinguish KD from other febrile illnesses. We did not refer to the recommendation of “at least 5 days of fever” and enable diagnosis earlier than medical experts using current KD diagnosis guidelines to suggest timely intervention. We developed a highly sensitive and specific algorithm for the diagnosis of KD. A prospective study of the laboratory variables in our model will be essential to determine its potential applications.

Several tests to diagnose KD have been developed. Ling et al. (29) reported one method, which involves combining clinical and molecular methods to distinguish KD from other febrile illnesses. That is the future research direction, but our diagnostic model did not include molecular methods. Such advanced technology must be validated in terms of its clinical value and if it is validated and practical, we will consider modifying our diagnostic algorithm by adding more sensitive and specific indicators. Maki et al. (30) reported a diagnostic scoring system using contrast-enhanced computed tomography (CT) findings for differentiating KD patients from children with other unexplained febrile illnesses and cervical lymphadenopathy. The sensitivity, specificity, and accuracy of their scoring system was 86%, 86%, and 86%, respectively, for diagnosing KD. The outstanding advantages of CT are high-density resolution, clear cross-section anatomy, and details of lesions, but it involves radiation exposure and is expensive. Ultrasound is non-invasive, does not involve radiation exposure, and is inexpensive. Therefore, from the perspective of safety and expense, our diagnostic model is more practical for clinicians and patients. In addition, enlargement of cervical lymph nodes is the least common feature of KD.

Independent predictors, such as the level of WBC, CRP, HB, %NEU, AST, ALT, TBL, albumin, and serum sodium, shown in previous diagnostic studies (17, 20, 21, 31) had a significant difference in the KD group and FCs group in our study. However, these predictors were not included in the final multivariate logistic regression model. In addition, the results of the univariate analysis may be different in various populations from different regions between the KD group and the FCs group. For example, the WBC level was significantly different between the KD group and FCs group in studies by Stemberger et al. (31), and Ling et al. (21), but not so in the study by Huang et al. (17). The CRP level was significantly different between the KD group and FCs group in our study and that of Song et al. (20), but not in the studies of Ling et al. or Stemberger et al. The serum level of chloride was significantly different between the two groups in our study, but not so in the studies of Stemberger et al. and Huang et al. This might be attributed to the fact that KD pathology is associated with genetic polymorphisms, and the genetic determinants of KD are different in various regions and populations, as reported elsewhere (32, 33). This difference might be related to the unknown etiology and genetic polymorphisms of KD, which can lead to different predictors for the KD diagnosis in different populations. Another possible reason for these discrepancies is the small number of patients studied and limited laboratory data. These differences might affect the difference between studies.

In our study, some new factors were significantly different between the KD group and FCs group: level of RBCs, RDWa, RDW, PCV, MPV, PDW, MCH, MCV, protein in urine, hematuria, AST, ALT, ALB, as well as serum levels of calcium, sodium, magnesium, and potassium. However, none of those factors were independent predictors. The urinary protein level in KD patients was much higher than that in FCs, which suggested that the function of glomerular vessels in KD patients was impaired. Muta et al. (34) reported that KD patients had a reduction in the serum level of sodium and phosphorus. We observed a significantly lower serum level of chlorine, phosphorus, potassium, magnesium, calcium, and sodium in the KD group, which suggested that kidney vasculitis might lead to adverse effects on tubular reabsorption and renal function. In addition, the increase in the level of GGT, ALT, DBIL, and TBIL, lower level of albumin and prealbumin, and the higher urinary level of bilirubin in the KD group might imply a more severe inflammatory reaction in the liver of KD patients (35).

We showed that age and the level of GGT, PLT, globulin, %MON, phosphorus, UA, %LYM, prealbumin, AST:ALT ratio, chloride, LDH were independent predictors for the diagnosis of KD. Among those predictors, studies have reported levels of PLT, P-LYM, GGT, and P-MON to be different (17, 21). An increased PLT is a characteristic feature of KD. In some studies, the degree of thrombocytosis was correlated with the risk of CAAs in KD. Durongpisitkul et al. (36) and Wang et al. (37) reported a reduction of %LYM in patients with KD, thereby suggesting a stronger inflammatory response. In this context, the GGT level in the KD group was much higher than that in the FCs group, a result which is in accordance with the data from a study by Tremoulet et al. (38) and Ting et al. (39). Tremoulet et al. (40) reported that the increased level of GGT was used to predict resistance to treatment with IVIG and an increased risk for CAAs. Age also plays a very important part in the clinical manifestations of KD. Stemberger et al. (31) have reported that age-related differences were present in the initial presentation of KD in a pediatric emergency department. Based on the individual predictors mentioned above, we established a new model for KD diagnosis with a sensitivity of 86%, a specificity of 81%, and an AUC of 0.907.

One of the strengths of our study was the use of routinely collected EMRs from a large dataset of KD patients and FCs over one decade. This sample size and number of items are much larger than those used in previous models for KD diagnosis. Another strength of the study was the use of FCs. For some febrile patients with a diagnosis of KD upon hospital admission, the diagnosis upon hospital discharge was febrile illness for which KD had been included in the differential diagnosis and who had a fever and at least one of the clinical features of KD. Our diagnostic algorithm for diagnosis in patients with KD may be used to help guide clinicians, especially in underdeveloped countries, in initial decisions about the stage of therapy.

Our study had four main limitations. First, a selection bias may have been present because our study was retrospective and from a single center. Second, some variables were not available, which might have led to a bias in statistical analyses. For data items with a missing detection rate <25%, we undertook MICE to reduce the risk of bias. Third, the treatment and assessment of patients were done by multiple clinical teams. Fourth, although all FCs had a standardized set of clinical laboratory tests for KD as recommended by pediatricians, very few FCs underwent echocardiography.

## Conclusions

This is the first study with large sample sizes to discriminate KD from other febrile illnesses in China. The diagnosis of KD could be predicted using age as well as the level of %MON, phosphorus, UA, globulin, %LYM, prealbumin, GGT, AST:ALT ratio, serum chloride, LDH, and PLT. Future prospective studies must be done to validate the utility of this new model and improve KD diagnosis.

## Data Availability Statement

The datasets generated for this study will not be made publicly available According to the Ethics Committee of the Children's Hospital Affiliated to Chongqing Medical University, we have been approved to use this part of clinical data for clinical research, but no permission has been granted for public inquiry and sharing.

## Ethics Statement

This study was approved by the Ethics Committee of the Children's Hospital Affiliated to Chongqing Medical University.

## Author Contributions

ZH designed the study, collected and analyzed the data, and drafted the initial manuscript. X-HT collected and analyzed the data. HW built the model and prepared all figures. BP edited the manuscript. T-WL and JT designed the study, reviewed, and edited the manuscript. All authors contributed to the article and approved the submitted version.

## Conflict of Interest

The authors declare that the research was conducted in the absence of any commercial or financial relationships that could be construed as a potential conflict of interest.
