# Supplementary material for: A New Diagnostic Model to Distinguish Kawasaki Disease From Other Febrile Illnesses in Chongqing: A Retrospective Study on 10,367 Patients
Source: Front Pediatr. 2020 Nov 12;8:533759. doi: 10.3389/fped.2020.533759 (PMC7693557; doi:10.3389/fped.2020.533759)
Supplement: Supplementary file 1 [file Data_Sheet_1.PDF]

Table1. The age distribution of three groups

Age	KD group (N=5642)	FCs group (N=4725)	incomplete KD group (N=809)
0-1 years	1225	1398	259
1-2 years	1518	861	209
2-3 years	1065	503	125
3-4 years	725	475	73
4-5 years	471	315	53
>6 years	638	1173	90

N: number of samples.

Table 2. Comparison of the incomplete-KD group and FCs group

Variable	Incomplete-KD group		FCs group	
	N	Mean±SD / Counts (%)	N	Mean±SD / Counts (%)
Platelet count, 10^9/L	697	440.90±195.43	4379	308.46±146.99
Percentage of lymphocyte	697	0.33±0.14	4379	0.42±0.20
Percentage of monocyte	693	0.04±0.02	4314	0.04±0.02
Gamma- glutamyl transpeptidase, U/L	791	47.83±68.40	4286	34.43±72.89
AST/ALT	791	1.61±0.90	4284	1.05±0.78
Lactic dehydrogenase, IU/L	791	299.02±116.25	4286	398.71±534.56
Prealbumin, mg/L	693	83.87±47.85	3694	124.88±54.58
Globulin, g/L	792	22.66±5.59	4289	21.57±6.02
Uric acid, umol	786	206.40±75.89	4074	259.66±115.96
Serum phosphorus, mmol/L	788	1.44±0.30	4131	1.50±0.35
Serum chlorine, mmol/L	788	102.26±3.37	4132	103.28±4.37
Age, month	809	28.92±28.14	4725	42.35±42.34

ALT: Alanine transaminase; AST: Aspartate aminotransferase; N: number of samples; SD: standard deviation.
